# Strengths, disconnects and lessons in local and central governance of the response to the first wave of COVID-19 in Ghana

**DOI:** 10.4314/gmj.v56i3s.10

**Published:** 2022-09

**Authors:** Lauren J Wallace, Nana E Enyimayew Afun, Anthony A Ofosu, Genevieve C Aryeetey, Joshua Arthur, Justice Nonvignon, Irene A Agyepong

**Affiliations:** 1 Dodowa Health Research Centre, Research and Development Division, Ghana Health Service, Accra, Ghana; 2 Ghana Health Service, Ministries, Accra, Ghana; 3 Department of Health Policy, Planning and Management, School of Public Health, University of Ghana, Accra, Ghana; 4 Public Health Unit, Komfo Anokye Teaching Hospital, Kumasi, Ghana; 5 Public Health Faculty, Ghana College of Physicians and Surgeons, Accra, Ghana

**Keywords:** COVID-19, Health Security, Governance, Ghana

## Abstract

**Objectives:**

To explore governance, coordination and implementation actors, structures and processes, facilitators, and barriers within local government and between central and local government in Ghana's COVID-19 response during the first wave of the outbreak.

**Design:**

Cross-sectional single case study. Data collection involved a desk review of media, policy and administrative documents and key informant in-depth interviews.

**Setting:**

Two municipalities in the Greater Accra region of Ghana

**Participants:**

Local government decentralised decision makers and officials of decentralised departments.

**Interventions:**

None.

**Main Outcome Measures:**

None

**Results:**

Coordination between the national and local government involved the provision of directives, guidelines, training, and resources. Most of the emergency response structures at the municipal level were functional except for some Public Health Emergency Management Committees. Inadequate resources challenged all aspects of the response. Coordination between local government and district health directorates in risk communication was poor. During the distribution of relief items, a biased selection process and a lack of a bottom-up approach in planning and implementation were common and undermined the ability to target the most vulnerable beneficiaries.

**Conclusions:**

Adequate financing and equipping of frontline health facilities and workers for surveillance, laboratory and case management activities, transparent criteria to ensure effective targeting and monitoring of the distribution of relief items, and a stronger bottom-up approach to the planning and implementation of interventions need to be given high priority in any response to health security threats such as COVID-19.

**Funding:**

This work is funded by International Development Research Centre Grant No. 109479, Exploring and learning from evidence, policy and systems responses to COVID-19 in West and Central Africa.

## Introduction

Public health emergencies are complex, and coordination between multiple levels of government and sectors is important for preparedness and response.^1^, ^2^ The International Health Regulations promote the participation of and coordination between central and local levels of government and all relevant sectors to prevent, detect and respond to public health emergencies of international concern. 1 Numerous global policy frameworks and resolutions related to health security in particular, and global public health, more broadly, as well as regional and sub-regional compacts, also highlight that health and its determinants are best addressed through the coordination between health and non-health sectors and actors.^3^,^4^,

Despite the necessity of good coordination, structuring, maintaining and sustaining coordination can be challenging.^5^,^5^,^6^,^7^

As urbanisation continues to increase globally, city-level governments, in particular, play an important role in responding to complex challenges such as health emergencies.^8^ More success has been attributed to approaches to governance that integrate the responses of multiple sectors, combine top-down and state-centric measures with community-based activities, and strong, democratic city-level governance.^10^ Different priorities between different levels of government, conflicts between actors, often over limited resources; and solely top-down and state-centric measures to direct and coordinate activities may contribute to the limited success of containing public health emergencies.^9^

Strong intra-organizational relations are critical for multisectoral action, and intra-government coordination, defined as coordination among public sector organisations (ministries, departments and agencies (MDAs)), is particularly important.^10^ Understanding how the coordination of the pandemic unfolds in specific intra-governmental contexts is useful for drawing lessons for preparedness, response and resilience for current and future health emergencies.

The general objective of this paper is to explore governance, coordination and implementation actors, structures and processes, facilitators, and barriers within local government and between central and local government during the first wave of Ghana's COVID-19 response and lessons for effective responses to health security threats in Ghana. We consider coordination a “process by which a public sector organisation endeavours that their actions take into consideration the activities, resources and outcomes of other organisations”.^11^, ^12^

## Methods

### Study design

The study design was a single cross-sectional case study. Our case definition was “Response and response coordination to COVID-19 at central and local government levels”.

### Study Setting

The Greater Accra Region of Ghana was purposively selected for this study as the region in Ghana that has had the highest number of COVID-19 cases and deaths.^13^ Greater Accra's Ashaiman and La-Nkwantanang Madina municipalities were purposively selected because of already ongoing work on health and urban poverty.

### Sources of data /Data collection

Data came from a desk review and twenty-three key informant in-depth interviews conducted in February and March 2021. Desk review focused on English language media and other grey literature (reports, briefing notes, strategies, guidelines, and others). Online portals of two newspapers (Daily Graphic, Daily Guide) and two radio stations (Citi News, My Joy Online) were searched using the Factiva (Dow Jones) database. The two newspapers and two radio outlets were selected based on the following criteria (1) a balance of public and private news outlets and (2) readership/popularity. The Daily Graphic is the most popular daily newspaper in Ghana, and the Daily Guide is a private newspaper with the second-highest readership. Citi FM and Joy FM rank in the top 5 most popular radio stations nationally. Factiva does not index any of Ghana's publically owned radio stations; however, most radio stations in Ghana (including all of the top 5) are private. Articles that contained Covid* or corona* in the title or in the lead paragraph for the first global wave of the pandemic (30^th^ January, when the WHO announced COVID-19 as a Public Health Emergency of International Concern through September 30^th^, 2020) were included. For reports, strategies and guidelines, the team compiled lists of government ministries, committees, bilateral and multilateral organisations, and non-governmental organisations (NGOs) involved in the COVID-19 response and searched their websites for relevant documents.

A total of 2586 (2521 media articles and 65 other documents) were retrieved. Eligibility of media reports and official documents for inclusion was determined by a quick scan of the report or executive summary, respectively, to determine if mention was made of the response of local government agencies to COVID-19 or processes of coordination/collaboration between authorities at various levels. A total of 2013 documents were excluded (1995 media reports and 18 other documents), with 573 (526 media reports and 47 other documents) included.

Ten interviews were conducted in Ashaiman and 13 in La-Nkwantanang Madina. Informants were selected purposively because of their role as key local government and district health system decision-makers and implementers in the COVID-19 response. Index respondents were the district health directors, with snowball sampling used to identify other relevant informants in the municipal health management team (MHMTs) and municipal assemblies (also known as Municipal, Metropolitan and District Assemblies or MMDAs). At the municipal Assembly, this included individuals such as assembly members, Municipal Chief Executives (MCEs), Coordinating Directors and heads of relevant departments. Respondents from the health system included officers in health promotion, surveillance, health information, disease control and case management.

Interviews were conducted over the telephone or zoom in English and lasted between 25 to 60 minutes. Participants were asked to describe the major actors involved in preparedness and response to COVID-19 in their municipality; the roles of the local government and the national governments and their perceptions of their effectiveness in the response; facilitators and barriers to coordinating key actors; and their recommendations for improving the response. Interviews were conducted by the first and second authors (LJW and NEA). Written consent was obtained from respondents, and permission for the study was given by the Ghana Health Service (GHS) Ethics Review Committee [no: GHS-ERC 012/10/20].

### Data analysis

Desk review data were coded by tagging relevant information by research question and cutting and pasting this information into a table together with the author, date, source and title. Interviews were manually coded using Excel^®^ by the first and second authors. They independently read and coded five interviews to ensure quality control in coding the interview data. Following the initial coding, they discussed their categories, codes and sub-codes, resolved discrepancies, and developed an agreed-upon code list. Themes and sub-themes were summarised and organised under relevant research questions using Microsoft Word^®^ and linked to representative quotes.

## Results

The findings have been structured based on our research objectives. The first section presents our findings on central (national) and sub-national (regional and municipal/district) government structures for leadership and governance of the response. The second section presents interventions for response planning and implementation and the roles of the national and sub-national governments for risk communication and other preventive protocols, case management, laboratory and surveillance, and relief programs. The final section describes the facilitators and barriers within local government and between the central and local governments that impacted the response.

### Structures for Leadership and Governance of the Response

#### National Level

Ghana's National Strategic COVID-19 Response Plan involves National-Level committees with clear roles and membership.^14^ It emphasises the “whole of the government and society” approach^15^ with clear multisectoral structures at all levels of government. Key National structures for the response to public health emergencies are the Inter-ministerial Coordinating Committee (IMCC), the National Technical Coordinating Committee (NTCC) and the Emergency Operations Centre (EOC). See Sarkodie and colleagues^16^ for a more fulsome description of National-Level committees.

### Local Government (Regional and District/Municipal) Levels

Public Health Emergency Management Committees (PHEMCs) were convened at regional and district levels. Regional PHEMCs were convened by the Regional Minister under the leadership of the Regional Coordinating Council (RCC) to oversee the regional response, with the Regional Minister as chairperson. Regional PHEMCs were representatives from GHS, health facilities, health promotion, disease control/surveillance, environmental health, and information services. and representatives from key agencies such as the Food and Drug Administration, National Disaster Management Organization (NADMO), Ghana Education Service (GES), Ghana Water Company, Medical services under military and police (quasi-government), faith-based organisations, such as churches and mosques and their associations (FBOs).

The MCE convened district/Municipal PHEMCs. PHEMCs had a diverse membership, including members from the MHMT (such as the municipal health director, health promotion officer, disease control officer and public health nurse); representatives of the MMDA such as the MCE, Coordinating Director and technical heads of key departments of the Assembly (including education, environmental health and sanitation, NADMO, and social protection and community development); and other stakeholders including representatives from the ambulance service, police, and fire service, health facilities and the Red Cross. Rapid response and case management teams were also convened at both the regional and municipal levels. Their roles and membership are discussed below under “case management, surveillance, and laboratory activities”.

Response planning and implementation processes focused on (1) Risk communication and other preventive protocols, (2) Surveillance, Laboratory and Case management, and (3) Relief programs.

### The roles of the national and sub-national government for risk communication and other preventative protocols

#### National Level

Ghana's first case of COVID-19 was confirmed on 12 March 2020. In response, the executive arm of the government set public health guidelines and issued directives to enhance mask wearing, social distancing, and hygiene. Beginning on 27 March, a three-week partial lockdown was implemented in parts of Greater Accra and Ashanti Regions, which were the epicentres of the first wave. An Executive Instrument (E.I.61), which declared COVID-19 a public health emergency was also implemented, which mandated mask wearing in all public places.^16^

### Local Government (Regional and District/Municipal Levels)

Municipal assemblies were responsible for: coordinating public education about COVID-19; multiple phases of a mass disinfection and fumigation exercise supported by Zoomlion (a private waste management company) in all public places, including markets, lorry parks, and schools; forming COVID-19 task forces to monitor and enforce adherence to preventative protocols; and market decongestion. Several national and sub-national actors were involved in COVID-19 public education, decongestion and disinfection exercises and the enforcement of preventative protocols in Ghana during the first wave of the pandemic. See (Table 1).

**Table 1:** Roles of national and sub-national actors in risk communication and other preventative protocols during the first pandemic wave in Ghana

National	
***National Government***	Issuing public health guidelines, legal frameworks, directives Presidential addresses to the Nation Financing Provision of logistics
***Ministry of Health & Ghana Health Service***	Press briefings, Issuing directives and guidelines, Media monitoring (GHS)
***Ministry of Information***	Press briefings, coordination of public education
***National Commission for Civic Education (NCCE)***	Coordination of public education
***Ministry of Local Government and Rural Development***	Coordination of market decongestion
***Ministry of Sanitation and Water Resources***	Coordination of mass disinfection/fumigation exercise
***Zoomlion***	Implementation of and (some) provision of funding for mass disinfection and fumigation exercise
***NADMO & security agencies (police, army, prisons service,*** ***etc.)***	Enforcement of public health directives
**Regional**	
***Regional Health Directorates***	Training MHMTs Provision of leaflets and other health promotion materials Linking MHMTs to media
***Regional Co-ordinating Councils***	Issuing and enforcing national directives/safety protocols
**Municipal/District**	
***MMDAs- Information services department***	Coordination of public education
***MMDAs- Environmental health department***	Coordination of mass disinfection/fumigation exercise & Market decongestion Formation of COVID-19 task forces for enforcement of preventative protocols
***MHMTs***	Coordination of public education
**Sub-municipal/district and community**	
***NADMO & security agencies (police, army, prisons service,*** ***etc.)***	Participation in municipal taskforces to enforce preventative protocols
***Market associations, FBOs, traditional authorities,*** ***NGOs, schools, health facilities, businesses, community*** ***health committees***	Educating memberships/communities on preventative protocols
***Market associations, FBOs, Traditional authorities***	Coordination of decongestion exercises
***Community information centres and media houses***	Public education

MHMTs and MMDAs provided education through myriad sources and strategies. One of the main strategies was a trainer of trainees approach in which education was given to assembly members, representatives of faith-based organisations such as leaders of churches, and other opinion leaders, representatives of NGOs, health facilities, as well as to heads of businesses, market leaders, schools, and transport operators. These representatives, in turn, educated their communities, and clients. Sensitisation was also carried out using information vans, radio broadcasts, and community information centers. Municipal assemblies took different approaches to market decongestion, including closing main markets and creating smaller satellite markets in outlying areas such as football fields, and shift systems. The roles of the national and sub-national government for case management, laboratory and surveillance activities

### The roles of the national and sub-national government for case management, laboratory and surveillance activities

#### National level

The implementation principles of the national COVID-19 response plan placed specific emphasis on a balance between decentralised and centralised responses to the pandemic, with a focus on national-level policies, guidelines and protocols as “essential to ensuring uniform standards of implementation” alongside “decentralisation of service delivery, testing, contact tracing and case management using the existing decentralised health systems of the GHS at Regional, District and Sub-district levels”.^17^ At the national level, the Noguchi Memorial Institute for Molecular Research (NMIMR), led initial testing for COVID-19 since it had the capacity and support to carry out complex virologic testing safely. Even though the National Public Health Reference Lab (NPHRL) is the designated lead for public health laboratory testing, the necessary capacity and logistics were not yet in place at the early stages of the pandemic. Under a Laboratory Quality Framework arrangement, capacity was built, first at the NPHRL, and later with other laboratories to widen the network of laboratories with the capacity to test for the SARS-CoV-2 virus safely. For a detailed list of various roles, see Table 2.

**Table 2:** Roles of national and sub-national actors in case management, laboratory and surveillance activities during the first pandemic wave in Ghana

National	
***National Government***	Financing Provision of logistics including vehicles
***Ministry of Health & Ghana Health Service***	Financing Provision of logistics including vehicles Expansion of infrastructure for testing, holding suspected cases, isolation of confirmed cases and treatment Training Issuing directives and guidelines
**Regional**	
***Regional Co-ordinating Councils***	Coordination of cross-sectoral response at the regional level Re-assignment of selected staff to treatment centres
***Regional Health Directorates***	Financing Technical coordination and support Training Provision of logistics and infrastructure Coordination of case management across facilities Coordination of laboratory results Convening regional case management teams Convening regional rapid response teams
* ***Laboratories (NMIMR, NPHRL, etc.)***	Molecular Testing for COVID-19 Training and capacity building for Zonal Reference Labs Establishing Laboratory Quality Framework Feedback of test results
**Municipal/District**	
***MMDAs***	Coordination of cross-sectoral response Financing Provision of logistics Facilitating the set-up of treatment and isolation centres Mobilising communities for COVID-19 testing Assisting contact tracers to navigate neighbourhoods
***MHMTs***	Convening and directing municipal rapid response teams Setting up treatment and isolation centres Convening and directing municipal case management teams Briefing MMDAs and the public Implementing guidelines/directives adapted to local situation Coordination of case management in facilities Re-assignment of selected staff to treatment centres Hiring contact tracers & Coordinating contact tracing Overseeing sample-taking; Packaging and transport of samples to designated labs
**Sub-municipal/district and community**	
***FBOs, NGOs, businesses, individual community members***	Donations of logistics to MMDAs, health facilities
***Health facilities and laboratories***	Management of cases Implementing guidelines

* Laboratory testing was initially led by the Noguchi Memorial Institute for Molecular Research at the national level. However, over time, due to the need to scale up testing capacity, training and capacity building took place for zonal reference labs such that COVID-19 testing was decentralised.

### Local Government (Regional and District/Municipal Levels)

MHMTs were a lead agency in the municipality for the COVID-19 response; however, they collaborated closely with the Assemblies. Respondents discussed two main roles of the MHMTs; first, they acted as the primary source of information about COVID-19 in the municipality. For instance, the municipal health director provided weekly briefings to the MMDAs and the MCE about COVID-19 prevention and transmission and the nature of the outbreak in the municipality. Second, MHMTs were responsible for coordinating case management in their municipality, including sample-taking and packaging and transporting samples to designated laboratories . MHMTs convened and directed Rapid Response Teams (RRT). Rapid Response Teams (RRTs) are technical, multidisciplinary teams meant to implement response measures in the early phase of an outbreak, but in the case of COVID-19, their activities became protracted and mostly merged with the case management team activities. Municipal RRTs are under the direction of their municipal PHEMCs and provide on-the-ground investigative support to inform decisions taken by the PHEMC. During the COVID-19 response, municipal RRTs in both municipalities included stakeholders such as the heads of major health facilities, the municipal director of health, health promotion officers, surveillance officers, biomedical scientists, disease control officers, health information officers and nurse managers. At the municipal level, case management teams were also formed to oversee the management of suspected and confirmed cases; these teams were comprised of diverse stakeholders, including clinicians, pharmacists, disease control officers, health information officers, mental health workers, and representatives from the national ambulance service. These teams communicated regularly through WhatsApp as well as Zoom. Municipalities had clinical leads who oversaw case management and helped triage patients to specific centers based on case severity and capacity at the treatment centers. Municipal case management teams also reported to and coordinated with a case management coordinator at the regional level. Municipal assemblies facilitated a number of case management and surveillance activities in collaboration with MHMTs (see Table 2).

### Relief programs

#### National Relief programs

The Government of Ghana implemented several interventions to mitigate the adverse socioeconomic effects of the pandemic and the pandemic response and to support adherence to public health directives. Water bills were absorbed for all Ghanaians from April to June 2020, and public and private water tankers were mobilised through a cost-rebate scheme to ensure water supply to all vulnerable communities. Electricity bills were absorbed for the poorest of the poor ('lifeline’ consumers), and for all other consumers, residential and commercial, 50% of the bill was absorbed over the same period. Other economic measures included a soft loan scheme for micro, small and medium-scale enterprises (MSMEs), a COVID-19 social support programme that disbursed cash grants to select extreme poor COVID-19-affected households, and the provision of dry and hot food packs to vulnerable persons within partial lockdown areas.^16^ Table 3 provides information on the roles of national and sub-national actors involved in social and economic relief programs during the first wave of the pandemic.

**Table 3:** Roles of national and sub-national actors involved in social and economic relief programs during the first pandemic wave in Ghana

National	
***National Government***	Issuing guidelines for food distribution Funding
***NADMO***	Sourcing and distribution of Food Relief Sourcing of water tankers
***Ministry of Gender, Children and Social Protection***	Coordination of Food Relief Coordination of social support program
***Ministry of Local Government***	Directives and Guidelines for water relief
***Ministry of Sanitation and Water***	Directives and Guidelines for water relief
***National Board for Small Scale Industries (NBSSI)***	Coordination of business loans for MSMEs
***Ghana Water Company***	Registration of water vendors for rebate
***Ghana Private Sector Fund***	Donations of food
	
***Ghana National Fire Service & Ghana Police Service***	Sourcing of water tankers
**Regional**	
***Regional Health Directorates***	Compiling lists of COVID-affected vulnerable persons for social support program
***Regional Co-ordinating Councils***	Issuing directives
**Municipal/District**	
***MMDAs- NADMO zonal officers***	Targeting/distribution of food relief
***MMDAs - Assembly members***	Targeting/distribution of food relief Assisting businesses with loan registration Sensitising water vendors and assisting them to register with Ghana water company Donations of food and money to communities
***MMDAs- Social welfare department***	Registration of individuals for social support program
***MHMTs***	Compiling lists of COVID-affected vulnerable persons for social support program Donations of food and money to COVID-affected vulnerable persons
***NBSSI Business advisory centres***	Registration of businesses for loans
**Sub-municipal/district and community**	
***Businesses, individual community members, and NGOs***	Donations of food
***FBOs***	Donations and distribution of food

### Facilitators and Barriers

#### Structures for Leadership and Governance of the Response

##### Local government (regional/municipal levels)

Most respondents indicated that the coordination of case management and surveillance within and between municipalities was effective due to the high technical knowledge of the staff of the MHMTs, RRTs and case management teams and their high level of dedication and motivation. Though the membership of the PHEMCs in the two municipalities was similar, some perceptions of functionality were different. In one municipality, some informants felt that the PHEMC was not fully functional and that there was an inadequate interest of the MMDA in the committee's work. The committee had been inaugurated but had not been meeting regularly.

### Coordination of Response Implementation

#### Risk communication and education

Respondents described some gaps in the coordination of public education, including inadequate resources for health promotion. Staff from the MHMT described how health communication activities require vehicles and funds for fuel and radio broadcasts. However, they had received insufficient resources, and as a result, the quality of COVID-19 education in their municipalities suffered. The Assembly provided a small amount of funds to the MHMTs at the start of the pandemic, but the members of the MHMTs were not clear on the details of the Assembly's use of the funds received from the national government other than the portion they were allocated.

Inadequate vehicles were an issue that impacted both the Assembly and the MHD. Both municipalities had received one vehicle from the national level during the first wave of the pandemic; however, these vehicles were later retrieved and no longer available for use. Beyond challenges with resources, members of the MHD perceived insufficient collaboration between the NCCE and the health promotion department of the MHD, negatively affecting public risk communication and education.

Members of the health promotion department suggested that this challenge resulted from the national government's decisions to empower the NCCE at the expense of health professionals who have the needed technical background.

Respondents described the coordination of the disinfection/fumigation exercises as effective. However, some respondents from one municipality felt that the market decongestion was poorly executed in their municipality since Assembly members were not involved in the planning process, and traders and the public were insufficiently educated about the intervention, which resulted in overcrowding. Further, in both municipalities, the activity of task forces was perceived to be inconsistent.

### Case management, surveillance and laboratory activities

A few respondents discussed challenges with communication surrounding case management between stakeholders at the municipal and regional or national levels. For example, after some remand prisoners tested positive in one municipality, there were delays in receiving directions. In both municipalities, delayed laboratory results slowed the effectiveness of case management and contact tracing. These delays were common at the start of the first wave and were thought to be due to limited laboratory capacity or to data entry issues resulting in a backlog of unprocessed samples.

Healthcare workers, RRTs and case management teams within the municipalities were viewed as highly capable. However, the implementation of planned case management and surveillance activities was negatively affected by limited resources. For instance, despite the fact the cost of the management of COVID-19 cases was to be borne by the national government, at the time of the study, health facilities managing COVID-19 cases had not yet received reimbursement for their expenditures for the management of cases. Municipalities also faced inadequate facilities for treatment, isolation and quarantine and an inadequate number of vehicles.

Regarding isolation facilities, respondents explained that there was a need for more isolation centres, especially since the municipalities were densely populated. Health facilities in the municipalities mostly had only holding areas for COVID-19-positive patients and were challenged by inadequate infrastructure and logistics, such as oxygen to manage severe or critical cases. Private health facilities participated in the local response. At the peak of the first wave, virtually all available beds to manage severe and critical cases in public facilities in the Greater Accra region became full. As a result, patients who could afford to pay for their own care were transferred to private facilities, which had become part of the regional network to scale up treatment capacity.

Respondents acknowledged that the budgets allocated to the region from the national level were inadequate, which negatively affected the response in municipalities. Some respondents from both MHMTs and assemblies felt that although national and regional budgets were inadequate, assemblies could have provided more resources to the MHMTs. The municipal assemblies received funds from the national government for the COVID-19 response through the Ministry of Local Government. The funds were earmarked for purchasing personal protective equipment (PPE), fumigation, and the provision of infra-structure for water relief, such as the construction of boreholes. The Common Fund secretariat also deducted part of the monies for good performance transferred from the common fund to well-performing assemblies. Leaders at the assemblies explained that inadequate funds had hampered the ability of the MMDA to respond effectively. In addition to the small budget allocated by the government to assemblies for the response, revenue collection had been negatively affected by the partial lockdown and other public health measures and the local and global economic disruption that had followed; as a result, the amount of internally generated funds the Assemblies had access to had decreased.

### Relief programs

Relief programs were useful; for instance, food relief reportedly reached thousands of families, electricity and water interventions had been financially beneficial to the public, and increased hygienic practices. Despite the large numbers of individuals and households reached, respondents, especially assembly members, expressed some dissatisfaction with the initiatives for various reasons. First, the selection and distribution processes were perceived to have been biased. Political affiliation with the ruling government was perceived to influence food distribution and the selection of the recipients of business loans. Political bias was particularly acute in food distribution. Respondents suggested that NADMO was not the ideal agency to coordinate food distribution.

The non-involvement of assembly members and the Assembly's department of social welfare contributed to the ineffective way food was shared, resulting in limited access to food by the most vulnerable. In the relief efforts of faith-based organisations such as Churches and Mosques, key informants did not observe the same extent of politicisation of relief distribution. FBOs seemed to be better able to deploy methods of reaching those who needed support in their constituencies which helped avoid discrimination.

Second, some relief programs had challenges with the registration and application process, including business loans, individual grants and loans, and water relief. Assembly members complained that there was no appropriate process or criteria to screen undeserving or unqualified loan applicants. Conversely, the loan application requirements (such as the need for a tax identification number) were perceived to exclude low-income individuals with the most vulnerable businesses and/or led to the payment of middlemen to apply.

Registration challenges also affected the water intervention. The Ghana Water company did not reimburse many private water vendors; as a result, vendors stopped providing the water for free. Some private water sellers, such as owners of mechanised boreholes with standpipes, refused to provide free water since they relied on immediate cash payments from sales revenue to finance routine expenses and could not afford to provide their services. Further, in urban areas, especially in informal settlements, households often rely on public bath facilities accessed for a fee; however, these facilities were not explicitly included in the free water guidelines.

## Discussion

The response of the government of Ghana to the COVID-19 pandemic was characterised by high-level political commitment and support for a whole-of-government and a whole-of-society approach, which bolstered coordination between health and non-health stakeholders. Coordination between the national and local government was in the form of national directives and guidelines, training, and provision of funds and logistics. The responses of local government seem to primarily have focused on implementing interventions identified by the national government. For the most part, clear directives, guidelines, and roles were given to local governments, with local-level actors, such as district assemblies, tasked with collaborating with specific ministries, departments and agencies to implement national interventions. Both municipal assemblies and MHMTs were key actors in response to COVID-19.

While MHMTs acted as the technical leads for the response and oversaw surveillance, testing and case management in municipalities, MMDAs convened and directed public health emergency management committees and coordinated the overall response (including the activities of diverse actors such as NGOs and FBOs, security agencies, businesses, water and sanitation companies, and transport operators). Studies suggest that governments often reactively consider mechanisms for multisectoral coordination of a public health emergency rather than before an event begins.^2^ This does not seem to be the case in Ghana; clear multisectoral governance structures were already set out to manage public health emergencies before the pandemic. Our findings suggest that most municipal structures were available and generally functional, with some exceptions, such as some municipal PHEMCs that did not meet regularly. Although this analysis focuses on the roles of, and coordination between, public sector organisations, the private sector and development partners played major roles in Ghana's response. For instance, the World Bank's COVID-19 Emergency Preparedness and Response Project committed USD 365 million in financing to the Government of Ghana.^17^

Inadequate resources, including funds and logistics, however, were a major challenge which cut across all aspects of the response. This challenge was felt acutely by frontline responders, who are key to any effective response. In particular, staff at treatment and isolation centres and contact tracing teams, the MHMT and the Assemblies responsible for risk communication and public education were hardest hit. Shortages of logistics, including PPE and laboratory reagents, and limitations in ICU bed space have also been reported by others.^18^,^18^ The inadequate resources may represent an inadequate national domestic emergency budget.^19^ Some respondents felt that despite limited budgets, municipal assemblies could have provided more resources to MHMTs.

Moreover, for some COVID-19 response activities, particularly risk communication, the Assembly and the MHMT were reportedly working in uncoordinated ways, which may have reduced the efficient use of limited resources. It is possible that the context of decentralisation in Ghana negatively influenced the coordination of some activities, such as health promotion during the emergency response. While health is administratively decentralised or deconcentrated, the local government, with many of its decentralised departments, has undergone devolution. This has resulted in dual lines of reporting and financing between the district assemblies and Ghana Health Service, with sometimes ad hoc and personality-dependent relationships between the MHMT and the district assembly.^20^

While the government of Ghana and other actors made considerable efforts to minimise the socioeconomic impacts of COVID-19, the implementation of relief interventions was reportedly marred by arbitrariness and the biased selection and distribution processes, as well as a lack of a bottom-up approach in planning and implementation. The politicisation of the distribution of relief items was most evident in food relief, in which politically exposed persons were in charge while others such as assembly members, were less involved. In their study of informal sector workers' experiences of lockdown restrictions in Kumasi, Adom and colleagues^21^ also uncovered the view that food relief was hoarded unjustly by the rich, and that needy people were neglected based on their political affiliations. The distortion of equity in the distribution of state programs and resources for political reasons is reported in the literature worldwide and not unique to COVID-19 or Ghana.^22^,^23^ Recognition of this possibility requires that for critical situations, it will be important to assign distribution decision-making and implementation to individuals and agencies who are relatively free from the pressures that lead to the politicisation of distribution.

In the case of loans for businesses, there seemed to be a lack of understanding by the NBSSI and other institutions involved of the needs of vulnerable groups and how to streamline the registration processes. Likewise, in the case of the free water directive, guidelines did not address some unique contexts of water insecurity in urban settings.^24^ One of the important roles of local governments is to be accountable to, and engage with urban residents through participatory forms of decision-making.^9^ Yet, the approach to the distribution of relief items seemed to be centralised, with the less-than-optimal engagement of communities and community leaders in planning and execution. This may also be a reflection of the politics of decentralisation, in which a centralising tendency towards the national government has persisted, despite supposed administrative, political and fiscal decentralisation of local governments.

This analysis offers a novel understanding of governance and coordination structures and processes within local government and between central and local governments in two select municipalities in Greater Accra during the first wave of the COVID-19 pandemic. However, the study has some limitations. The findings from only two municipalities may not be generalisable to all localities in Greater Accra. Second, while sampling knowledgeable stakeholders reflect their lived experiences with the COVID-19 pandemic, it is not possible to rule out the potential for reporting bias; purposive sampling may have introduced bias since the sample may not be representative of all knowledgeable stakeholders.

In addition, the selection of only 4 of the most popular online news sources may not reflect the true reality of the first wave of the pandemic. Despite these methodological limitations, the news sources selected had a large readership. The triangulation of multiple sources of information (interviews, media, and documents at the national and district levels) may mitigate selection bias and improve the validity of the results.

## Conclusion

Despite the high commitment of the government of Ghana to a whole of government and a whole of society approach, as stated in national strategies and presidential speeches, and many strengths such as high-quality staff and established plans, local governance of the pandemic was challenged in several ways. Some of these challenges, such as incomplete decentralisation, slow funds transfer, and poor equipping of health facilities, reflect pre-existing governance and logistical challenges and reinforce the need to address them. We also recommend effective monitoring of the distribution of relief items, a bottom-up approach to the planning and implementation of relief interventions, and the identification of additional sources/mechanisms of financing public health emergencies at all levels.
